# Virtual Reality Simulation for Undergraduate Nursing Students for Care of Patients With Infectious Diseases: Mixed Methods Study

**DOI:** 10.2196/64780

**Published:** 2025-02-11

**Authors:** Wen Chang, Chun-Chih Lin, Julia Crilly, Hui-Ling Lee, Li-Chin Chen, Chin-Yen Han

**Affiliations:** 1 Department of Nursing Chang Gung University of Science and Technology Taoyuan City Taiwan; 2 Nursing Management Department of Administration Center Chang Gung Medical Foundation Taoyuan City Taiwan; 3 Department of Nursing Chang Gung University of Science and Technology Chiayi County Taiwan; 4 Department of Nursing New Taipei Municipal TuCheng Hospital (Built and Operated by Chang Gung Medical Foundation) New Taipei City Taiwan; 5 Department of Emergency Medicine Gold Coast Health Southport Australia; 6 School of Nursing and Midwifery Griffith University Southport Australia

**Keywords:** virtual reality, infection control, learning motivation, learning attitudes, nursing education

## Abstract

**Background:**

Virtual reality simulation (VRS) teaching offers nursing students a safe, immersive learning environment with immediate feedback, enhancing learning outcomes. Before the COVID-19 pandemic, nursing students had limited training and opportunities to care for patients in isolation units with infectious diseases. However, the pandemic highlighted the ongoing global priority of providing care for patients with infectious diseases.

**Objective:**

This study aims to (1) examine the effectiveness of VRS in preparing nursing students to care for patients with infectious diseases by assessing its impact on their theoretical knowledge, learning motivation, and attitudes; and (2) evaluate their experiences with VRS.

**Methods:**

This 2-phased mixed methods study recruited third-year undergraduate nursing students enrolled in the Integrated Emergency and Critical Care course at a university in Taiwan. Phase 1 used a quasi-experimental design to address objective 1 by comparing the learning outcomes of students in the VRS teaching program (experimental group) with those in the traditional teaching program (control group). Tools included an infection control written test, the Instructional Materials Motivation Survey, and a learning attitude questionnaire. The experimental group participated in a VRS lesson titled “Caring for a Patient with COVID-19 in the Negative Pressure Unit” as part of the infection control unit. In phase 2, semistructured interviews were conducted to address objective 2, exploring students’ learning experiences.

**Results:**

A total of 107 students participated in phase 1, and 18 students participated in phase 2. Both the VRS and control groups showed significant improvements in theoretical knowledge scores (for the VRS group *t*_46_=–7.47; *P*<.001, for the control group *t*_59_=–4.04; *P*<.001). However, compared with the control group, the VRS group achieved significantly higher theoretical knowledge scores (*t*_98.13_=2.70; *P*=.008) and greater learning attention (*t*_105_=2.30; *P*=.02) at T1. Additionally, the VRS group demonstrated a statistically significant higher regression coefficient for learning confidence compared with the control group (β=.29; *P*=.03). The students’ learning experiences in the VRS group were categorized into 4 themes: Applying Professional Knowledge to Patient Care, Enhancing Infection Control Skills, Demonstrating Patient Care Confidence, and Engaging in Real Clinical Cases. The core theme identified was Strengthening Clinical Patient Care Competencies.

**Conclusions:**

The findings suggest that VRS teaching significantly enhanced undergraduate nursing students’ infection control knowledge, learning attention, and confidence. Qualitative insights reinforced the quantitative results, highlighting the holistic benefits of VRS teaching in nursing education, including improved learning outcomes. The positive impact on student motivation and attitudes indicates a potentially transformative approach to nursing education, particularly in the post–COVID-19 era, where digital and remote learning tools play an increasingly vital role.

## Introduction

### Background

Virtual reality (VR) has emerged as an innovative teaching strategy in nursing education. VR technology leverages simulated scenarios to overcome time and space limitations, offering students opportunities to learn in safe, realistic settings and receive immediate feedback [1]. VR simulation (VRS) teaching strategies enhance learning motivation, student immersion, knowledge and skill acquisition, confidence [2,3], active participation, and learning effectiveness [4-6]. The goal of undergraduate nursing education is to prepare students for clinical practice, making it essential to strengthen their professional competencies and attitudes. Integrating information technology into nursing education enhances students’ learning outcomes. Nursing education should align with the broader clinical practice environment, incorporating technology to support students in developing their competencies [7].

The COVID-19 pandemic has profoundly impacted nursing curricula and teaching worldwide. In emergency and critical care, university-level nursing curricula must reflect clinical environments. Emphasizing situated learning enhances students’ abilities and confidence in providing emergency patient care [8,9]. Before the pandemic, nursing students rarely had opportunities to care for patients with infectious diseases in isolation units. However, the demand for care related to infectious diseases remains a global priority [9]. Strengthening courses on infectious diseases can help students develop positive attitudes toward clinical practice [10]. Updating infectious disease courses with more practical experiences can further support nursing students in developing positive attitudes when caring for patients with infectious diseases during clinical practice.

Learning theories related to VR teaching include constructivism, situated learning, and experiential learning. In VR learning, learners actively absorb information and construct new knowledge [11]. Situated learning theory emphasizes real-world interactions and activities in authentic contexts, transforming these experiences into applicable knowledge [12]. VR offers an interactive virtual environment, using visual effects to present abstract problems and providing opportunities for active manipulation and repeated practice [11]. Experiential learning theory posits that learning is the transformation of experience, with knowledge creation emerging from interactions, conflicts, and problem-solving between individuals and their environment. This theory highlights the potential of immersive technology to provide meaningful experiences [12]. Compared with other teaching methods, VR teaching is easy to use, generates positive and active learning experiences [13], and enhances learning outcomes, including improvements in knowledge, skills, and clinical decision-making [14,15]. Engagement in VR environments provides students with experiences closely aligned with clinical practice, boosting their motivation and attitudes and leading to better educational outcomes [14,15].

Motivation and attitude play a significant role in influencing learning outcomes. Enhanced motivation strengthens active learning and improves results [12,16]. Studies have shown a positive correlation between motivation and learning outcomes, making learning easier and fostering proactive engagement [17,18]. Keller’s Attention, Relevance, Confidence, and Satisfaction (ARCS) model of motivation incorporates a learning motivation scale to assess motivational aspects within a course [12,16]. Designing courses with integrated motivational models can inspire learners, enhance motivation, and increase classroom engagement [19,20]. In nursing education, particularly in emergency and critical care courses, VR can address the limitations of clinical settings and traditional teaching methods caused by resource constraints [21-24]. VR stimulates learners’ motivation, promotes active participation, and enhances learning outcomes [25,26]. By incorporating VR teaching, courses can more closely align with clinical practice, providing students with a solid foundation in professional knowledge and skills.

Even long after the pandemic, there will remain a global need for the care of patients with infectious diseases. However, opportunities for students to participate in the actual care of such patients in isolation units remain limited. To date, little attention has been given in the literature to identifying educational strategies that address this gap in developing nursing students’ professional knowledge and skills. This mixed methods study was guided by 2 research questions: (1) What is the effectiveness of VRS teaching on nursing students’ theoretical knowledge, learning motivation, and attitudes toward the care of patients with infectious diseases? and (2) What are the learning experiences of nursing students in a VRS program? Our a priori hypothesis was that VRS teaching would significantly improve nursing students’ infection control theoretical knowledge, learning motivation, and attitudes regarding the care of patients with infectious diseases.

### Objectives

This study had 2 objectives: (1) to evaluate the effectiveness of VRS teaching on nursing students’ theoretical knowledge, learning motivation, and attitudes toward the care of patients with infectious diseases, and (2) to explore their learning experiences in a VRS program designed for this target population.

## Methods

### Study Design

This study used a 2-phased mixed methods approach to comprehensively evaluate a VRS teaching program on the care of patients with infectious diseases, which was part of the infection control unit within the Integrated Emergency and Critical Care course. Phase 1 utilized a quantitative study design to assess the learning effectiveness of the VRS teaching method, while phase 2 used qualitative phenomenography to explore students’ experiences and perceptions of the program.

### Phase 1: Outcomes of the VRS Program on Students’ Knowledge, Learning Motivation, and Attitudes to the Care of Patients With Infectious Diseases

#### Overview

A quasi-experimental design was used to compare learning outcomes—knowledge, motivation, and attitude—between students in the VRS teaching program (experimental group) and those in the traditional teaching course (control group). Data were collected from August 2022 to July 2023.

#### Participants

This study used convenience sampling and was conducted at a clinical competence center at a university in Taiwan. Third-year undergraduate nursing students enrolled in the Integrated Emergency and Critical Care course were eligible to participate. One class of students was assigned to the experimental group, and another to the control group. The Integrated Emergency and Critical Care course is an elective offered in both the first and second semesters. Researchers used a random selection process to assign students in the infection control unit to the VRS program in the first semester and to traditional teaching in the second semester. The participating school provided a 2-week add/drop period, during which members of the research team gave in-class briefings about the study, and students were free to choose whether to participate in the experimental group. The selection criteria for the experimental group were (1) aged ≥20 years, (2) enrolled in the Integrated Emergency and Critical Care course, and (3) willing to participate in this study. Students with a history of epilepsy were excluded from the VRS. Sample size estimation was conducted using G*Power software version 3.1 [27]. Following Cohen’s rule [28], 2 groups were included, with a medium effect size of *f*=0.25, a correlation of 0.5, a power of 0.8, and an α value of .05, resulting in a required sample size of ≥86, with ≥43 participants per group. A total of 47 students were recruited for the experimental group. None of the students in the experimental group refused to participate in the VRS program. All participants were taught by the same instructor, and the course content was consistent across both groups.

#### Instruments

##### Infection Control Written Test

Previous research has shown that VRS teaching can enhance the development of both knowledge and practical skills in undergraduate nursing students, with outcomes effectively assessed using a written test [29]. In this study, the infection control knowledge assessment involved a written test administered to students before (T0) and after (T1) the infection control lesson. The test consisted of 10 questions aligned with the learning objectives of the infection control unit. These included single- and multiple-choice items covering both theoretical knowledge and practical skills, such as donning and doffing personal protective equipment (PPE). The test addressed the same key infection control techniques for all students, aiming to evaluate their baseline abilities and the changes in knowledge following the lesson. To better capture postlearning changes and minimize the influence of memory recall on the posttest results, the order of the questions was adjusted, and some questions were modified. The test items were reviewed by the course instructor and clinical experts (senior emergency nurses) to ensure content validity.

##### Instructional Materials Motivation Survey

The Instructional Materials Motivation Survey (IMMS) is a self-reported questionnaire administered before (T0) and after (T1) the infection control lesson. Designed primarily to evaluate students’ motivation in learning a course [12], the IMMS comprises 36 items distributed across 4 subscales based on the ARCS motivation model: Attention (12 items), Relevance (9 items), Confidence (9 items), and Satisfaction (6 items). Each item is rated on a 5-point Likert scale, with higher scores indicating greater learning motivation. The original IMMS scale has demonstrated high reliability, with Cronbach α values ranging from 0.81 to 0.96 [12]. In this study, the IMMS exhibited excellent reliability, with a Cronbach α of 0.94.

##### Learning Attitude Questionnaire

A learning attitude questionnaire was administered at T0 and T1. This 20-item self-reported questionnaire was developed by several members of the research team to assess students’ attitudes toward caring for patients with infectious diseases and their participation in the infection control unit. Items were rated on a 5-point Likert scale, where 1 indicates “strongly disagree,” 2 “disagree,” 3 “neutral,” 4 “agree,” and 5 “strongly agree.” Higher scores reflected a more positive learning attitude. The questionnaire demonstrated strong validity and reliability, with an average Content Validity Index of 0.9 and a Cronbach α of 0.955.

##### VRS Lesson Plan for Caring for a Patient With COVID-19 in the Negative Pressure Unit

**Figure 1:**
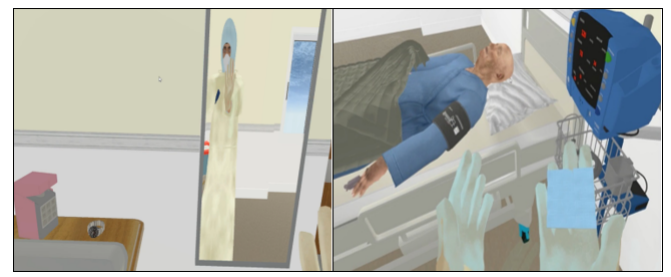
Screen captures of virtual reality simulation videos.

In the infection control unit, VRS teaching was implemented for the experimental group. The VRS scenario, developed by several research team members with VR training certification, depicted a real clinical case of a febrile patient visiting the emergency department for triage, later confirmed to have COVID-19, and subsequently admitted to a negative pressure isolation unit (Figure 1). The teaching content emphasized a nurse’s role in providing care within a negative pressure isolation room, including the proper techniques for donning and doffing PPE. The lesson’s learning objectives were for students to differentiate care for patients with infectious diseases, correctly don and doff PPE, and provide appropriate patient care. The VR Oculus Quest equipment, including a headset and controllers, was supplied by the School of Nursing of the participating university. The VRS lesson plan was reviewed by the course instructor and clinical experts (senior emergency nurses) to ensure content validity. The lesson utilized VR technology to deliver immersive visual effects and interactive scenarios, aiming to enhance students’ awareness and provide opportunities for practical exercises, thereby improving learning outcomes [30]. The assessment included the standard procedure for applying PPE, such as N95 masks, goggles, hair caps, and gloves. Upon completing the assessment, students received immediate feedback, with the computer screen highlighting missed items. This direct feedback was intended to reinforce learning effectiveness [1].

#### Procedure

**Figure 2:**
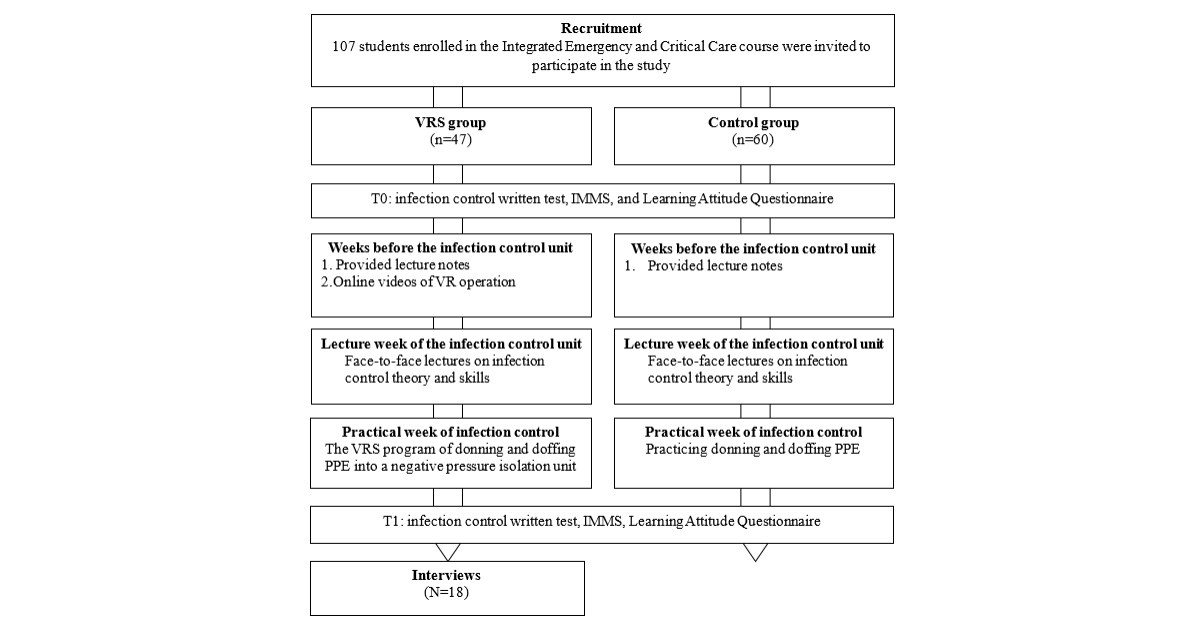
Study protocol process. IMMS: Instructional Materials Motivation Survey; PPE: personal protective equipment; VR: virtual reality; VRS: virtual reality simulation.

To maintain neutrality, none of the research team members were involved in teaching either the experimental or control group. Instead, several team members focused on designing the VRS program and creating a VR system operation video to help students become familiar with operating the VR system. During the experimental group’s class, research team members were available to address any technical issues that participants encountered. They also met with the unit instructors before the start of the unit to ensure consistency in teaching between the 2 groups and alignment in the course delivery process. The study protocol is illustrated in Figure 2.

The infection control unit spanned 2 weeks and included 1 lecture (100 minutes) and 1 practical session (100 minutes). Both the VRS group and the control group received lecture notes before the start of the course. Additionally, the VRS group was provided with prerecorded online VR videos demonstrating donning and doffing PPE in a negative pressure isolation unit, as well as instructions on operating the VR system. For the VRS group, the first week consisted of a 100-minute lesson on infection control theory and skills, while the second week involved 100 minutes of VRS instruction. The VRS class was divided into 5 groups, each comprising 8-10 students, who worked collaboratively on the drills. The lesson began with an introduction to VR system operation (5-10 minutes), followed by group VRS scenario drills (30-40 minutes). Each student then executed their part of the VRS lesson, which lasted approximately 6-8 minutes. Upon completion, the system provided feedback, serving as the students’ learning outcomes. Group members first discussed the session among themselves, followed by a 10-minute instructor-led debriefing session. During this session, students were encouraged to ask questions and share their reflections on the VRS program execution. Feedback and reflection were incorporated to help students consolidate their learning and transform it into meaningful learning outcomes. The groups then switched roles and conducted a second round of drills and discussions for another 30-40 minutes. A pretest (T0) and posttest (T1) on infection scenario cases were conducted to evaluate the students’ learning outcomes, motivation, attitudes, and knowledge related to the infection control unit. For the control group, a traditional teaching strategy was used. During the first week, theoretical lectures were delivered, accompanied by a video to aid students in understanding the process. Lecture notes, identical to those provided to the VRS group, were distributed before class and included a video link demonstrating the standard PPE procedure. In the second week, students were divided into 6 groups of 9-11 members to practice donning and doffing PPE. Instructors provided individualized guidance to correct mistakes. Within the same groups, students evaluated and discussed the PPE practice. Although an instructor-led debriefing session was planned for the control group, it was postponed to the following week due to the large number of students and time constraints.

Data collected for this study were individually coded and entered into a computer for analysis using SPSS version 22.0 (IBM Corp.). Descriptive statistics, including frequency, percentage, mean, and SD, were calculated. Inferential statistics, such as independent *t* tests, paired *t* tests, and generalized estimating equations, were applied. Results with a *P* value of <.05 were considered statistically significant.

### Phase 2: The Students’ Learning Experiences of the VRS in Caring for Patients With Infectious Diseases

#### Overview

Phase 2 utilized qualitative phenomenography to explore students’ experiences and perceptions of the VRS program. The key concepts in phenomenography are “phenomenon” and “experience.” This methodology aims to identify the shared and generalized aspects of participants’ thoughts or concepts regarding their experiences of a specific phenomenon, with a focus on describing their understanding of these experiences [31]. In this study, phenomenography was applied to understand how learners organize and structure the content they acquire during the learning process [31]. Interviews with students were analyzed to uncover their learning experiences and outcomes, with the goal of providing evidence to support the ongoing improvement of educational programs.

#### Participants

Students in the experimental group who met the following inclusion criteria were recruited: (1) aged 20 years or older, (2) enrolled in the Emergency and Critical Care course and participating in VRS teaching, and (3) consenting to participate in and have interviews recorded.

#### Procedure

Participants took part in in-depth semistructured interviews. These interviews facilitate meaningful conversations, providing a detailed understanding of complex issues [31]. In a phenomenographic study, interview questions need to be as open-ended as possible to accurately capture the participants’ thoughts. The interview guide is provided in Multimedia Appendix 1. The in-depth interviews in this study contributed to understanding nursing students’ experiences with VRS teaching. Each eligible participant received a consent form outlining the study’s purpose, the voluntary nature of participation, and the confidentiality of their data. Participants completed the consent forms, and suitable interview times were scheduled. The interviews were conducted by a single researcher (CYH) who had prior experience in qualitative research gained during doctoral studies, had served as a principal investigator on research projects, and had published several qualitative research articles. CYH was not involved in teaching this subject and was unfamiliar with the participants in the experimental group. During the interviews, CYH utilized interview skills to encourage participants to articulate their VR learning experiences. The interviews were audio-recorded and lasted between 42 and 62 minutes. A sample size of 18 students was sufficient to generate rich data and achieve saturation.

### Qualitative Analysis and Trustworthiness

Following each interview, the same researcher (CYH) transcribed the audio recordings verbatim to ensure detailed documentation and analyzed the interview data. Data analysis followed the 7 steps of phenomenographic analysis: familiarization, condensation, comparison, grouping, articulating, labeling, and contrasting [32]. The trustworthiness of the research findings was established using Lincoln and Guba’s [33] criteria of credibility, transferability, dependability, and confirmability. In terms of credibility, phenomenographic research emphasizes the precise description of each stage of the study process, the application of the researcher’s ideas to the phenomena, the careful formulation of interview questions and processes, and the thorough analysis and presentation of conclusions. Peer debriefing, which involves collaborative data analysis to explore diverse interpretations, enhances data interpretation and credibility, contributing to the development of credible research outcomes. Transferability is supported by providing in-depth data that represent a comprehensive view of the research, highlighting its relevance and context. Dependability is ensured by supporting categorizations with excerpted interview content, illustrating the similarities and differences among participants in relation to the phenomenon, and confirming the logical connection between the collected data and the phenomena captured by the descriptive categorization. Confirmability is established by documenting the interviewer’s feelings and thoughts during the interview process, thereby creating an audit trail. The data analysis is thoroughly described, with detailed records of decisions made and strategies adopted during concept formation. These reflections on theoretical and methodological aspects further contribute to the audit trail and the confirmability of the findings [34].

### Ethical Consideration

Ethical approval was obtained from the Institutional Review Board of Chang Gung Medical Foundation (approval number 202002386B0). Potential participants were fully informed about the nature and purpose of the study, emphasizing that participation was entirely voluntary and that they had the right to withdraw from the study at any time. They were explicitly assured that their academic results would not be affected by their decision to participate or not. Participants were also guaranteed that their data would remain confidential and that they would not be identifiable in any reports. All participants provided written informed consent, and none of the students withdrew from the study.

## Results

### Phase 1: Outcomes of the VRS Program on Students’ Knowledge, Learning Motivation, and Attitudes to the Care of Patients With Infectious Diseases

#### Overview

Participants in phase 1 consisted of 107 third-year undergraduate students: 47 in the experimental group, who received VRS teaching, and 60 in the control group, who participated in traditional practical sessions on donning and doffing isolation gowns. As shown in Table 1, the majority of participants (99/107, 92.5%) were female, with an average age of 21.14 (SD 0.69) years.

**Table 1:** Demographic characteristics of participants in phase 1 (N=107).

Participant demographics		Study groups		Total participants (N=107)
		Virtual reality simulation (n=47)	Control (n=60)	
**Gender, n (%)**				
	Male	2 (4.3)	6 (10)	8 (7.5)
	Female	45 (95.7)	54 (90)	99 (92.5)
**Age (years), mean (SD)**		N/A^a^	N/A	21.14 (0.69)
	Male	22.00 (1.41)	20.50 (0.55)	20.88 (0.99)
	Female	21.40 (0.54)	20.96 (0.70)	21.16 (0.67)

^a^N/A: not applicable.

#### Effectiveness of VRS in Infection Control Theoretical Knowledge

**Figure 3:**
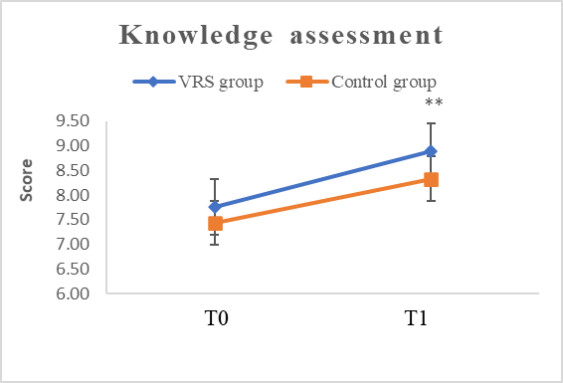
Change in infection control knowledge in the two groups. T0: pretest; T1: posttest; **: *P*<.01.

Pre- and posttest assessments of infection control knowledge were conducted at T0 and T1, with a maximum score of 10 points. The combined results of all participants showed an average pretest knowledge score of 7.58 (SD 1.13) and a posttest knowledge score of 8.58 (SD 1.16), indicating improved knowledge after course completion (*t*_106_=–7.08; *P*<.001). Both the VRS and control groups demonstrated significant improvements in theoretical knowledge scores (for the VRS group *t*_46_=–.747; *P*<.001 and for the control group *t*_58_=–4.04; *P*<.001). However, the VRS group achieved significantly higher posttest scores compared with the control group (*t*_98.13_=2.70; *P*=.008), suggesting that VRS teaching was more effective in enhancing students’ knowledge (Table 2 and Figure 3).

**Table 2:** Comparison of theoretical knowledge scores in the 2 groups (N=107).

Variable				T0^a^, mean (SD)		T1^b^, mean (SD)		*t* test (*df*)		*P* value
**Knowledge assessment**										
	Virtual reality simulation			7.77 (1.05)		8.89 (0.79)		–7.47 (46)		<.001
	**Control**			7.43 (1.18)		8.33 (1.34)		–4.04 (58)		<.001
		*t* test (*df*)	1.52 (105)		2.70 (98.13)		N/A^c^		N/A	
		*P* value	.13		.008^d^		N/A		N/A	
Total				7.58 (1.13)		8.58 (1.16)		–7.08 (106)		<.001

^c^N/A: not applicable.

#### Effectiveness of VRS on Learning Motivation

The learning motivation of all students increased slightly from T0 (mean 3.84, SD 0.47) to T1 (mean 3.94, SD 0.40) (*t*_106_=–3.10; *P*=.002), with no significant differences between the groups at either T0 (*t*_76.50_=0.09; *P*=.93) or T1 (*t*_80.95_=1.43; *P*=.16). At T0, except for the Confidence dimension—which was lower in the VRS group compared with the control group (*t*_78.53_=–2.12; *P*=.04)—the other dimensions of the ARCS model (ie, Attention, Relevance, and Satisfaction) did not differ significantly between the groups. At T1, only the Attention dimension differed significantly between the VRS and control groups, being higher in the VRS group (*t*_105_=2.30; *P*=.02), as shown in Table 3.

**Table 3:** Comparison of learning outcomes between the 2 groups at different time points (N=107).

Variable and groups			T0^a^							T1^b^				
			Mean (SD)		*t* test (*df*)		*P* value		Mean (SD)			*t* test (*df*)		*P* value
**Motivation**					0.09 (76.50)		.93					1.43 (80.95)		.16
	Virtual reality simulation	3.84 (0.57)						4.01 (0.46)						
	Control	3.83 (0.38)						3.89 (0.34)						
**Attention**					0.91 (105)		.36					2.30 (105)		.02^c^
	Virtual reality simulation	3.84 (0.67)						4.03 (0.52)						
	Control	3.74 (0.48)						3.82 (0.42)						
**Relevance**					0.76 (82.99)		.45					0.88 (105)		.38
	Virtual reality simulation	4.15 (0.54)						4.21 (0.48)						
	Control	4.07 (0.41)						4.14 (0.37)						
**Confidence**					–2.12 (78.53)		.04^c^					0.86 (105)		.39
	Virtual reality simulation	3.45 (0.58)						3.65 (0.49)						
	Control	3.66 (0.40)						3.58 (0.43)						
**Satisfaction**					0.51 (78.30)		.61					0.48 (105)		.63
	Virtual reality simulation	3.98 (0.64)						4.18 (0.51)						
	Control	3.92 (0.44)						4.13 (0.50)						
**Attitude**					–0.60 (105)		.55					0.03 (105)		.98
	Virtual reality simulation	4.09 (0.57)						4.34 (0.52)						
	Control	4.15 (0.50)						4.34 (0.56)						

^a^T0: pretest.

^b^T1: posttest.

^c^*P*<.05.

To address potential bias stemming from the differences in the Confidence dimension between the VRS and control groups at T0, the Generalized Estimating Equations model was applied to analyze and compare changes in both groups throughout the study period and to evaluate the outcomes of the VRS intervention. For the VRS group, the regression coefficients for the Confidence dimension were significant (β=.29; *P*=.03), with positive parameter estimates compared with the control group. This finding indicates that the VRS intervention enhanced students’ learning confidence.

#### Effectiveness of VRS on Learning Attitude

The learning attitude score increased slightly in the VRS group from T0 (mean 4.09, SD 0.57) to T1 (mean 4.34, SD 0.52) and in the control group from T0 (mean 4.15, SD 0.50) to T1 (mean 4.34, SD 0.56). However, no significant differences were observed between the groups at either T0 or T1, as shown in Table 3.

### Phase 2: The Students’ Learning Experiences of the VRS in Caring for Patients With Infectious Diseases

#### Overview

In phase 2 of this study, 18 students from the VRS group who had expressed willingness to be interviewed were recruited for qualitative interviews. All interview participants were female. Data analysis followed the phenomenographic steps of familiarization, condensation, comparison, grouping, articulating, labeling, and contrasting [32]. Each theme elicited from the participants’ pool of meaning represented a concept of their learning experiences associated with engaging in the VRS program. The core theme captured the relationship between each theme and participants’ overall understanding of their VRS learning experiences. The students’ learning experiences were categorized into 4 themes: (1) Application of Professional Knowledge to Patient Care, (2) Enhanced Infection Control Skills, (3) Demonstrated Confidence in Patient Care, and (4) Participation in Real Clinical Cases. The core theme was identified as Strengthening Clinical Patient Care Competencies.

#### Theme 1: Application of Professional Knowledge to Patient Care

The students described how they applied their theoretical knowledge of infection control during the VRS teaching process, particularly regarding the various factors that need to be considered when entering and exiting the negative pressure isolation unit. Through the feedback and debriefing provided by the VRS teaching, they were able to reflect on the content of their infection control learning, thereby deepening their professional knowledge in this area.

#### Theme 2: Enhanced Infection Control Skills

The participants shared their experiences of using VRS to practice the care skills learned in the infection control unit. Through hands-on practice and observing their classmates, they noted improvements in their skills. They also reported that the course’s practical and interactive scenarios enhanced their learning interest, which translated into positive learning outcomes.

#### Theme 3: Participation in Real Clinical Cases

Most of the students in the integrated care course for Emergency and Critical Care expressed a desire to intern or work in emergency departments or intensive care units. However, during the pandemic, many hospitals’ critical care units halted the acceptance of nursing interns or prevented students from participating in the care of patients with infectious diseases during their ward internships. The participants reported that the realistic, scenario-based case studies in the VRS enabled them to practice clinical skills that would otherwise have been unavailable, thereby bridging the gap between theory and practice. The experience of providing care for simulated patients in a context closely resembling clinical settings enhanced their learning experience.

#### Theme 4: Demonstrated Confidence in Patient Care

During the interviews, participants shared that they had been concerned about their ability to provide effective clinical care for patients with infectious diseases due to the impact of the pandemic. However, after participating in the VRS course, they reported a boost in their confidence in providing this type of care.

#### Core Theme: Strengthening Clinical Patient Care Competencies

The core theme that emerged from the analysis of the qualitative data on students’ learning experiences in the VRS teaching program was the strengthening of their clinical care competencies. The VRS learning program allowed students to apply the professional knowledge and skills they had learned in the course to carefully design patient scenarios. Through VRS practical exercises, students improved the skills required to care for patients with infectious diseases. By engaging with clinical cases and performing learning tasks in a realistic setting, they gained greater confidence in caring for patients with infectious diseases. In other words, this approach of connecting learning experiences enhanced their clinical care competence, better preparing them for the future care of patients with infectious diseases.

## Discussion

### Principal Findings

The results of this study showed significant improvements in infection control knowledge scores in both groups, with the VRS group achieving higher scores, highlighting the effectiveness of VRS teaching in enhancing theoretical knowledge. The VRS group also achieved a higher attention score at T1 compared with the control group. Additionally, the VRS intervention enhanced students’ learning confidence. Students’ reflections on their learning experiences and perceptions of the VRS teaching emphasized the following themes: Application of Professional Knowledge to Patient Care, Enhanced Infection Control Skills, Demonstrated Confidence in Patient Care, Participation in Real Clinical Cases, and Strengthening Clinical Patient Care Competencies.

This study made every effort to control variables to ensure consistency in learning content and teaching quality between the 2 groups, including the use of the same teaching materials and the same instructor for both groups. The VRS provided immediate feedback, allowing students to actively engage in the care process within a VR patient scenario, which contributed to enhanced learning outcomes in the VRS group. However, due to the larger number of students in the control group, their debriefing session was delayed. Future studies could investigate the impact of debriefing timing on the effectiveness of VRS-based teaching.

### Comparison With Prior Work: Effectiveness of VRS in Infection Control Knowledge

The results of this study demonstrated significant improvement in infection control knowledge in both groups after the learning process. The VRS group achieved significantly higher scores on the infection control written test compared with the control group at T1, indicating that VRS teaching was more effective in enhancing students’ theoretical knowledge. This finding aligns with previous research. Systematic reviews and meta-analyses on VR in nursing education have demonstrated its effectiveness in improving knowledge [5,35]. Another review reported a moderate effect size (*g*=0.47) for VR teaching in knowledge acquisition [29]. Additionally, this review noted that subgroup analysis showed VR training involving multiple self-practice sessions of less than 30 minutes was effective in imparting procedural knowledge to undergraduate nursing students [29]. This finding is consistent with the results of our study, where each student engaged in VRS learning for 6-8 minutes, with the option for continued practice for those wishing to further develop their skills. An integrative review also concluded that VRS teaching is effective in enhancing the acquisition of clinical skills and knowledge [36]. An extensive review of 29 randomized controlled trials involving 2722 students found that VR, augmented reality, and mixed reality were as effective as traditional methods in enhancing knowledge, highlighting their potential role in preclinical education [37]. Similarly, a German study on teaching tracheal suction skills observed no statistical differences among various teaching methods in terms of knowledge and skill improvement, suggesting that VR can serve as a supplementary resource to existing learning strategies, supporting students in preparing for clinical practice [23].

### Impact of VRS on Learning Motivation and Attitude

A systematic review and meta-analysis of 26 studies found no significant impact of VR on nursing students’ motivation and cognitive load compared with traditional teaching methods [38]. This aligns with the findings of our study, which also showed no significant difference in learning motivation. However, other studies have reported higher motivation and satisfaction with VR, though it may also increase cognitive load [39]. Additionally, several studies have shown that VR positively impacts learners by enhancing attention and motivation, building self-efficacy, and reinforcing learning confidence and performance [5,6,21,25]. A Taiwanese study comparing traditional and VR teaching on nasogastric tube feeding found nonsignificant higher scores in the VR group, which demonstrated greater motivation and satisfaction but also experienced a higher cognitive load [39]. These findings highlight the need to carefully consider cognitive load in future course designs [39].

A South Korean study reported higher neonatal resuscitation knowledge, motivation, problem-solving skills, and confidence in the VR group compared with the control groups, along with lower anxiety levels [26]. An integrative review on VR teaching for emergency patients also revealed increased confidence in handling emergencies [25]. Although some studies have reported no significant differences in anxiety and confidence [3,40], further research is needed to determine VR’s impact on learning confidence and stress. A Chinese study on disaster nursing courses found significant improvements in preparedness, confidence, and performance in the experimental group, highlighting VR’s potential as a cost-effective simulation method [41]. Technical issues with VR were noted as disadvantages, which may explain the lower precourse confidence in the VRS group compared with the control group. Ensuring that students are familiar with the VR system before the course begins may help improve their confidence [1,27,42,43].

### Student’s Learning Experiences of VRS

Analysis of the qualitative data obtained in this study revealed 4 themes in students’ experiences and perceptions of VR learning: Application of Professional Knowledge to Patient Care, Enhanced Infection Control Skills, Demonstrated Confidence in Patient Care, and Participation in Real Clinical Cases. The core theme identified was the Strengthening Clinical Patient Care Competencies. Similarly, previous qualitative studies have demonstrated a positive impact of VR learning on knowledge [23,24,43], skills [21,23,24], confidence [23], and engagement [4]. Some of these studies focused on VR learning as a tool, while others examined the characteristics of the VR environment [21,23,43]. By contrast, our study applied a phenomenographic methodology to explore students’ experiences and perceptions of VR learning, linking these to various outcomes. As a result, our findings provide unique insights into students’ conceptions of VR learning.

### Strengths and Limitations

The strength of this study lies in its combination of quantitative and qualitative methods, providing a comprehensive understanding of the effectiveness of VRS teaching. However, some potential limitations and weaknesses should be considered. Although the sample size was adequate, it may not fully represent the diversity of nursing students, as it was drawn from only 1 university. The course in which the VRS program was applied was an elective unit offered in both the first and second semesters, with a maximum enrollment of 60 students per class. The number of students enrolled in each class was beyond the researchers’ control, resulting in an imbalance in the number of students between the 2 groups. It is recommended that future studies compare the effectiveness of VRS using groups of equal size and a larger number of participants. This study did not conduct a formal survey on potential side effects among students in the experimental group. However, during the VRS session, research team members and the instructor periodically checked in with the students. None of the students reported any discomfort that required them to pause or stop the activity. The control group engaged in practical exercises for donning and doffing PPE, which differs from the traditional nursing classroom teaching methods used in previous studies. Future research is needed to build on our findings and develop a more detailed understanding of the effectiveness of VRS programs.

### Conclusions

This study highlights the effectiveness of VRS teaching in enhancing infection control knowledge, learning motivation, attitudes, and course satisfaction among undergraduate nursing students. By combining insights from qualitative data with quantitative information, we have provided a holistic understanding of the potential role of VRS in nursing education. Despite its limitations, this study opens avenues for future research and presents a compelling case for the broader implementation of VR in nursing education curricula. Future studies should consider longitudinal designs to evaluate the long-term impacts of VRS teaching on nursing education. Additionally, expanding the participant pool to include a more diverse range of students could yield more generalizable results. The findings have significant implications for nursing education, suggesting that VRS teaching can effectively enhance learning outcomes, particularly in areas that require high levels of practical knowledge and skills. The positive impact on student motivation and attitudes also points to a potentially transformative shift in how nursing education can be delivered, especially in a post–COVID-19 era, where digital and remote learning tools are becoming increasingly important.

### Use of Generative Artificial Intelligence

During the preparation of this work, the authors used ChatGPT (OpenAI) to enhance the clarity of the content. After using ChatGPT, the authors reviewed and edited the content as needed and took full responsibility for the content of the published article.

## Appendix

Multimedia Appendix 1 Interview guide.

## Abbreviations

ARCS: Attention, Relevance, Confidence, and Satisfaction
IMMS: Instructional Materials Motivation Survey
PPE: personal protective equipment
VR: virtual reality
VRS: virtual reality simulation

## Data Availability

The data that support the findings of this study are available on reasonable request from the corresponding author.
