# Supplementary material for: Virtual Reality Simulation for Undergraduate Nursing Students for Care of Patients With Infectious Diseases: Mixed Methods Study
Source: JMIR Med Educ. 2025 Feb 11;11:e64780. doi: 10.2196/64780 (PMC11862763; doi:10.2196/64780)
Supplement: Multimedia Appendix 1 [file mededu_v11i1e64780_app1.pdf]

Appendix: Interview Guide

1. Could you talk about why you chose to take the Integrated Emergency and Critical Care course?
2. Could you talk about your experiences or process in taking the Integrated Emergency and Critical Care course?
3. Could you talk about the process of participating in the VR simulation of caring for a patient with COVID-19 in the Integrated Emergency and Critical Care course?
4. What did you learn from participating in the VR simulation of caring for a patient with COVID-19 in the Integrated Emergency and Critical Care course?
5. Could you talk about anything you feel impressive from participating in the VR simulation of caring for a patient with COVID-19 in the Integrated Emergency and Critical Care course?
6. How do you think participating in the VR simulation of caring for a patient with COVID-19 in the Integrated Emergency and Critical Care course differed from other courses?
7. What impact do you think participating in the VR simulation of caring for a patient with COVID-19 in the Integrated Emergency and Critical Care course would have on your future ability?
8. Do you have anything else you would like to share?
